# Printing to Fight Antimicrobial Resistance Through Nano/Microstructured Platforms

**DOI:** 10.1002/tcr.70186

**Published:** 2026-06-05

**Authors:** Giorgia Puleo, Silvia Orecchio, Dario Savoca, Claudia Pellerito, Vittorio Ferrara, Vincenzo Piscopo, Sebastiano Alberto Fortuna, Alessandra Giardina, Bruno Pignataro, Paola Costanzo, Floriana Campanile, Giuseppe Arrabito

**Affiliations:** ^1^ Department of Physics and Chemistry ‐ Emilio Segrè University of Palermo Palermo Italy; ^2^ Department of Biological Chemical and Pharmaceutical Sciences and Technologies (STEBICEF) University of Palermo Palermo Italy; ^3^ Istituto Zooprofilattico Sperimentale della Sicilia Palermo Italy; ^4^ Department of Biomedical and Biotechnological Sciences, Section of Microbiology University of Catania Catania Italy; ^5^ Department of Biotechnological and Applied Clinical Sciences (DISCAB) University of L’Aquila L’Aquila Italy; ^6^ Department of Chemistry and Chemical Technologies University of Calabria Rende Italy

**Keywords:** additive manufacturing, antimicrobial resistance, chemically engineered interfaces, nanoscale confinement, stimuli‐responsive materials

## Abstract

Antimicrobial resistance is one of the most pressing global public health threats, responsible for millions of deaths annually. To tackle this issue, additive manufacturing has been increasingly exploited to fabricate structured antimicrobial platforms with precise control over composition, geometry, and release kinetics. This review covers the most relevant approaches across different scales, from dip‐pen nanolithography and inkjet printing to 3D and 4D printing, emphasizing the role of nanoscale confinement, microenvironmental control, and interfacial chemistry as key design parameters. Polymeric matrices, metal and metal oxide nanostructures, and theranostic platforms are discussed in relation to their fabrication‐dependent properties, including material stability, ageing, and translational challenges. Finally, stimuli‐responsive architectures, phototherapeutic strategies, and artificial intelligence‐driven design are outlined as emerging tools toward next‐generation antimicrobial materials.

## Graphical abstract

Additive manufacturing enables the rational design of structured antimicrobial platforms across multiple length scales, from nanofibrous scaffolds and droplet microreactors to 3D and 4D printed constructs. By engineering confinement, interfacial chemistry, and stimuli‐responsive release, these systems exert localized action through complementary mechanisms against resistant pathogens.

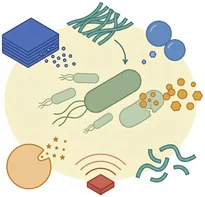

## 1 Introduction

Antimicrobial resistance (AMR) represents one of the most significant chemical and medical challenges of the 21^st^ century, driven by the widespread and often uncontrolled use of antibiotics and biocidal agents across clinical, industrial, and environmental settings. As conventional systemic treatments lose efficacy or induce severe cytotoxicity, the focus of research has shifted toward localized, precision‐engineered solutions. This transition moves beyond simple drug administration to the design of microstructured material platforms capable of navigating the complex chemical interface between therapeutic agents and microbial targets [1]. In this emerging paradigm, antimicrobial efficacy is no longer regarded as an intrinsic property of a molecular agent alone, but rather as an emergent outcome arising from the chemical composition, structure, and organization of the material platform in which that agent is incorporated.

This conceptual shift moves beyond traditional drug delivery approaches toward the rational engineering of structured materials capable of modulating the physicochemical microenvironment at the bacteria–material interface. At this interface, local parameters such as concentration gradients, diffusion rates, surface energy, redox activity, and molecular stability play a decisive role in determining whether bacterial adhesion, proliferation, or inactivation occurs. Consequently, controlling these parameters through material design offers a powerful route to enhance antimicrobial performance while reducing overall chemical load and mitigating off‐target toxicity.

The following sections focus on the chemical principles that govern how tailored material architectures translate into antimicrobial functionality. Differently from other reviews that focus on the emerging 3D‐printing materials against bacterial biofilm [2] or on electrospinning techniques [3] for antibacterial polymeric coatings, herein the printing methodologies discussed herein are unified by the chemical motifs of confinement, transport, and stability. By structuring materials at the micro‐ and nanoscale, it is possible to engineer specific microenvironments that control the diffusion of antimicrobial agents, protect labile molecules from degradation, and enhance oxidative pathways against biofilms. This review aims to provide a comprehensive framework for understanding how the spatially controlled chemical design of structured materials can be leveraged to create the next generation of AMR‐fighting technologies. In this landscape, the emerging role of generative artificial intelligence (AI) in the de novo design of AMPs and in the data‐driven optimization of printing ink formulations represents a critical upstream complement to the fabrication strategies discussed herein, opening new avenues for the fully integrated, computationally‐guided design of antimicrobial platforms.

## 2 Production and Chemical Functionalization of Biomaterials Across Length Scales

**FIGURE 1:**
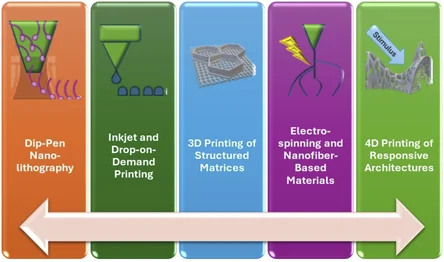
Novel biomaterial additive manufacturing techniques addressing antibacterial activity.

The antimicrobial performance of structured materials is intimately linked to the way they are fabricated. In this context, fabrication technologies should not be viewed as neutral or purely technical steps, but rather as chemical tools that define how antimicrobial microenvironments are created, maintained, and evolve over time. This section explores the spectrum of fabrication technologies enabling the design of antimicrobial platforms across multiple length scales. The transition from conventional manufacturing to high‐resolution printing and spinning methods allows for the strict modulation of surface topography, porosity, and chemical gradients at the specific length scales of bacterial interactions. This structural control enables the design of interfaces that actively interfere with bacterial colonization and biofilm formation by dictating molecular transport and interfacial reactivity. The following subsections analyze how these diverse fabrication strategies (Figure 1 and Table 1) are utilized to enhance antimicrobial efficacy through targeted material design.

**TABLE 1:** Comparison of fabrication techniques discussed in this review for the production of structured antimicrobial platforms.

Technique	Underlying principle	Typical resolution	Key advantages for antimicrobial applications	Representative antimicrobial materials
DPN	Capillary/diffusive ink transfer via AFM tip	50–100 nm (down to sub‐50 nm)	Nanoscale chemical patterning; reduced material load; precise surface control	TiO_2_ patterns; AMPs; metal oxide microdomains
Inkjet and Drop‐on‐Demand (DOD) printing	Controlled droplet ejection (thermal or piezoelectric actuation)	20–100 µm	Spatial control over chemistry; tunable droplet microenvironments; device customization	TCDMDA polymer matrices; metal oxide inks; AgNPs and ZnO suspensions
3D printing (FDM, SLA, SLS)	Layer‐by‐layer extrusion or photopolymerization	10–50 µm (SLA/DLP); 50–300 µm (FDM)	Controlled porosity and ion release; composite design; hierarchical scaffold architecture	PLA, PCL, PLGA composites with AgNPs, ZnO, TiO_2_, CuO
Electrospinning	Electric‐field‐driven fiber formation from polymer solution	50 nm–1 µm	High surface area‐to‐volume ratio; sustained release; tunable fiber morphology	PCL/SF fibres with AgNPs; curcumin‐loaded fibres; ciprofloxacin‐loaded PBS scaffolds
4D printing	Stimuli‐responsive shape or property change upon fabrication	50–300 µm	On‐demand release; adaptive response to infection‐associated triggers (pH, temperature, light)	PLA and PU, shape‐memory polymers; responsive hydrogels; EGaIn hybrid systems
5D printing	Multiaxis deposition along curved and nonplanar trajectories	50–300 µm	Biomimetic anisotropic architectures; curvature‐driven composition gradients; complex antimicrobial constructs	Emerging antimicrobial micro‐ and nanorobotic systems
Microcontact printing (µCP)	Stamp‐based pattern transfer via elastomeric molds	1–10 µm	Simple patterning; surface functionalization without high temperatures	SAMs; antimicrobial coatings; protein patterns
Aerosol jet printing	Aerosolized ink focused by gas stream	10–50 µm	Fine features on nonplanar substrates; nano‐spike surface morphologies	Metal nanoparticles; conductive antimicrobial inks
Screen/gravure printing	Paste forced through mesh screen or engraved cylinder	10–500 µm	High throughput; thick‐film deposition; scalable coatings	Metal oxide pastes; antimicrobial functional films
Liquid metal (LM)‐mediated printing	Spontaneous surface oxidation of LM precursors	10–50 µm	Conformal deposition; self‐healing; flexible substrate compatibility	Ga_2_O_3_ and In_2_O_3_ oxide films; reconfigurable antimicrobial coatings

### 2.1 Dip‐Pen Nanolithography

Dip‐pen nanolithography (DPN) represents the extreme limit of fabrication‐driven chemical control, operating at length scales where confinement itself becomes a dominant chemical variable, exploiting an atomic force microscopy (AFM) tip to generate “attoliter reactors” via capillary transport or diffusive flow. In the context of antimicrobial materials, the relevance of DPN has been used to create patterns of TiO_2_ and other metal oxides on biomedical metal surfaces, such as stainless steel 316L and titanium. The combination of DPN with soft lithography techniques, including microstamping, enables the replication and transfer of nanometric patterns onto extended surfaces. These patterned oxide domains act as chemically and topographically programmed interfaces, where photocatalytic activation and surface energy modulation jointly suppress bacterial adhesion. Studies have demonstrated that micropatterned surfaces with photocatalytically activated TiO_2_ significantly reduce adhesion of *S. mutans* and other pathogens, suggesting promising applications in dental and orthopedic contexts [4].

Beyond simple pattern definition, DPN enables the formation of surface‐bound microdomains and molecular condensates that precisely regulate physicochemical interactions at the bacterium–material interface. This level of spatial control allows antimicrobial performance to be achieved with substantially reduced material load, addressing toxicity issues often associated with bulk nanomaterials. Arango‐Santander and colleagues demonstrated the use of DPN and soft lithography to engineer material surfaces through well‐defined micropatterns intended for antibacterial applications. In their study, titanium dioxide was deposited with high spatial precision, allowing them to lower the TiO_2_ loading required to suppress the viability of adhering bacteria to ≈5% by weight. Their results highlight how nanoscale patterning can enhance photocatalytic efficiency and reduce the overall material demand while maintaining antimicrobial performance [4]. Building on these topographical and chemical cues, recent investigations have expanded the scope of scanning probe lithography (SPL) beyond simple inorganic deposition to complex mechano‐bactericidal surfaces. The precise engineering of nanoscale topography, which can be achieved via lithographic methods, can induce sufficient mechanical stress when it is in contact with the bacterial envelope to cause lysis, independent of leaching chemicals [5]. SPL techniques are pivotal in this domain as they allow for the decoupling of surface chemistry from topography, enabling the rational design of multifunctional interfaces that combine physical rupture with chemical attack. This versatility is particularly relevant for the controlled grafting of antimicrobial peptides (AMPs). Recent studies emphasize that the efficacy of AMPs is strictly dependent on their surface density and orientation; lithographic deposition provides the necessary control to tether these labile molecules in optimal conformations, maximizing membrane disruption while minimizing host cytotoxicity [6]. Furthermore, the rapid emergence of AI in de novo drug design has created a bottleneck in experimental validation. The concept of miniaturized arrays has also been translated into peptide and biomolecular microarrays in other contexts, where high‐density arrays are used for parallel interrogation of thousands to millions of unique sequences. For example, scalable peptide microarray platforms have been developed that display millions of peptides per slide and have been applied to simultaneous detection and classification tasks, underscoring the utility of dense, spatially segregated biomolecule arrays for high‐throughput screening and interaction profiling [7].

### 2.2 Inkjet and Drop‐on‐Demand Printing

Inkjet and DoD printing technologies operate in the femtoliter‐to‐picoliter range, where each droplet produced acts as a transient microreactor [2]. In these confined droplets, solvent evaporation and solute transport are regulated by evaporation‐driven flows, generating highly controlled droplet microenvironments [8]. Rapid solvent loss induces local supersaturation, resulting in nonequilibrium self‐assembly, phase segregation, and the formation of chemically distinct microdomains [9]. This process allows for the precise and specific deposition of desired materials, including bioactive agents, and is particularly useful for the fabrication of antimicrobial devices [2].

The formulation of stable and printable inks is a critical step in this process, as ink rheology and interfacial properties directly determine droplet formation, stability, and final chemical outcome. Parameters such as viscosity, surface tension, and particle or precursor concentration must be carefully balanced to ensure reliable droplet formation and deposition, while simultaneously preserving the chemical integrity of antimicrobial components. In DoD printing, droplets are generated on demand using an external actuation mechanism that produces a pressure pulse in the ink chamber. Droplet volumes typically range from a few to tens of picoliters, allowing precise control of film thickness and resolution. This mechanism can exploit the thermal effect, in which localized heating expels the droplet. Although simple, this method is not suitable for thermolabile materials. A second mechanism that can be exploited is the piezoelectric process, in which pressure‐induced piezo deformation causes the droplets to eject, producing more uniform films suitable even for sensitive materials, making them more relevant for antimicrobial applications.

A representative example of the potential of inkjet‐based approaches was reported by He et al., who employed inkjet 3D printing with photopolymerizable formulations based on tricyclodecane dimethanol diacrylate (TCDMDA) for the fabrication of customized medical devices capable of resisting biofilms [9]. Rapid UV‐polymerization following droplet deposition allowed for the maintenance of complex geometries, while also providing antimicrobial capacity throughout the polymer matrix, dramatically reducing *Pseudomonas* *aeruginosa* biofilm formation compared to standard polymers. Notably, this strategy achieved biofilm resistance without relying on antibiotics, highlighting how ink formulation and droplet‐level chemistry can be leveraged to obtain antimicrobial performance. A related example highlighting the ability of printing techniques to control the spatial distribution of bioactive molecules is provided by Vigni et al., who developed polybutylene succinate (PBS) scaffolds loaded with BMP‐2 for bone regeneration in a rabbit model [10]. This study demonstrates how controlled printing and scaffold microarchitecture regulate the presentation and biological effectiveness of embedded active agents. From a materials chemistry perspective, metal oxide inks used in inkjet and DoD printing can be broadly divided into nanoparticle‐based suspensions and molecular precursor solutions. Nanoparticle inks offer the advantage of preformed crystalline material, potentially reducing the thermal budget required for device fabrication, but face challenges related to particle aggregation, sedimentation, and the need for effective necking or sintering to achieve continuous, conductive films. Precursor‐based inks, conversely, can form dense, homogeneous films but typically require higher processing temperatures to drive complete decomposition of organic ligands and crystallization of the oxide phase [11, 12].

Inkjet printing and DoD printing thus combine mechanical precision and chemical control, converting femto‐ or picoliter‐sized droplets into microreactors capable of producing defined structures. Combined with the formulation of versatile inks and controlled ejection processes, these techniques enable the production of uniform, customizable films and complex biomedical devices with specific characteristics, including antimicrobial properties.

### 2.3 Three‐Dimensional Printing of Structured Matrices

Unlike nanoscale patterning and droplet‐based printing, 3D printing enables antimicrobial functionality to be engineered through continuous 3D architectures in which porosity, connectivity, and polymer network chemistry govern molecular transport and ion diffusion [13, 14]. 3D printing techniques such as fused deposition modelling (FDM), stereolithography, and selective laser sintering (SLS) techniques allow the incorporation of metallic nanoparticles into biocompatible thermoplastic polymers such as polylactic acid (PLA), poly(ε‐caprolactone) (PCL), and poly(lactic‐co‐glycolic acid) (PLGA). During printing, nanoparticles of Ag, Cu, ZnO, and TiO_2_ retain their structural and functional properties, resulting in a homogeneous distribution within the polymer matrix [13]. The antimicrobial performance of these composites is governed by the interplay between particle dispersion, polymer chemistry, and matrix porosity. For example, PLA‐based composites loaded with silver nanoparticles (AgNPs) at 5–10 wt% exhibit antimicrobial efficacy exceeding 99% against E. coli and S. aureus, with suitable mechanical properties for biomedical use [15]. Beyond material composition, 3D technology also enables precise control of porosity and surface topography, which are critical factors for modulating bacterial adhesion and controlled release of metallic ions. By tuning printing parameters and infill geometry, it is possible to regulate diffusion pathways and local concentration gradients within the scaffold, thereby influencing both short‐term bactericidal activity and longer‐term release profiles [16, 17].

In systems designed to encapsulate biologically active antimicrobial agents, the printable formulation is referred to as a bioink, a polymer‐based matrix that must simultaneously satisfy rheological requirements for deposition and preserve the activity of encapsulated agents such as peptides, proteins, or bioactive nanoparticles throughout fabrication [18]. The development of functional bioinks represents a primary challenge in current 3D printing technologies, requiring a careful balance between processability and the preservation of biological efficacy. In particular, effective bioinks for antimicrobial platforms require crosslinking approaches that preserve the structural and functional integrity of the encapsulated agents. Traditional chemical methods often rely on reactive species or UV irradiation that can induce cytotoxicity or denature biomolecules such as proteins. To this end, the Amino Acid Decorated and Light Underpinning Conjugation Approach (ANADOLUCA) method represents a noninvasive photochemical strategy that employs ruthenium‐based amino acids to induce covalent crosslinking in proteinaceous matrices via photoelectron transfer under visible light, maintaining protein conformation and activity without UV exposure [19]. Another versatile approach involves enzymatic crosslinking, where biocatalysts such as microbial transglutaminase or horseradish peroxidase facilitate stable covalent network formation under physiological conditions, maintaining the functional integrity of encapsulated agents. Microbial transglutaminase induces crosslinking between the ε‐amino groups of lysine and the carboxylic acid groups of glutamic acid, forming stable protein‐based polymeric networks [20], while horseradish peroxidase tunes the hydrogel's mechanical properties through oxidative coupling of phenolic residues in the presence of hydrogen peroxide, yielding a crosslinked network compatible with encapsulated biological agents [21]. Other strategies are based on ionic interactions and complex coacervation, avoiding the use of toxic chemical reagents. Ionic crosslinking with multivalent cations induces rapid gelation of anionic polymers such as alginate or gellan gum at physiological pH, while complex coacervation exploits the entropic gain associated with counterion release upon association of oppositely charged polyelectrolytes [22, 23].

Alongside these approaches, programmable biomolecular glasses have recently been identified as a promising class of printable matrices for antimicrobial applications. Unlike traditional glasses characterized by irreversible covalent or ionic networks, materials such as biomolecular noncovalent glasses and high‐entropy cyclic peptide (HECP) glasses are stabilized by multivalent, reconfigurable hydrogen‐bonding networks [24, 25]. This noncrystalline yet dense molecular organization confers high mechanical integrity, with stiffness reaching up to 18.2 GPa, optical transparency exceeding 97%, and exceptional resistance to enzymatic hydrolysis in physiological environments where conventional polymers often fail. These properties make biomolecular glasses suitable candidates for the incorporation of diverse antimicrobial agents, including metal ions, AMPs, and small‐molecule drugs, within a protective matrix that shields labile cargos from degradation [25]. HECP glasses have demonstrated the ability to protect sensitive therapeutics from enzymatic degradation in simulated gastric and intestinal fluids, significantly extending their functional lifespan [24]. The supramolecular plasticity of these systems enables processability via extrusion‐based 3D printing under mild conditions, facilitating the fabrication of complex, high‐surface‐area architectures designed to maximize interfacial contact with microbial targets. Finally, the sluggish molecular diffusion within these hyperconnected networks allows for tunable controlled release profiles, programmable by modulating the hydration state or precursor stoichiometry to sustain effective local antimicrobial concentrations over time.

### 2.4 Electrospinning and Nanofiber‐Based Materials

Electrospinning has emerged as one of the most chemically versatile techniques for producing nano‐ and microfibrous architectures, owing to its ability to translate polymer solutions, dissolved in highly volatile solvents, into continuous fibres through solvent evaporation under a strong electric field [26]. As the charged jet travels from the needle toward the collector, elongation, thinning, and rapid evaporation give rise to nonwoven meshes with exceptional surface area, tunable porosity, and chemically adjustable interfaces [27].

Such electrospun nanofibrous materials offer a unique fabrication route within materials science, because their high surface‐to‐volume ratio, tunable porosity, and structural similarity to the extracellular matrix provide properties that are difficult to achieve with bulk materials [28, 29]. In the context of antibacterial applications, these fibrous scaffolds emerge as ideal candidates for wound dressings, patches, and on‐demand drug‐release systems. For instance, in studies on diabetic wounds, researchers fabricated electrospun silk fibroin or PCL nanofibers loaded with curcumin or other bioactive compounds, observing sustained release, enhanced tissue repair, and antibacterial activity against both Gram‐positive and Gram‐negative bacteria [30, 31, 32].

Moreover, the versatility of electrospinning allows the incorporation of inorganic antimicrobial nanoparticles or metal‐based agents: AgNPs‐based nanofibers demonstrated broad‐spectrum bactericidal activity, combining the mechanical properties of synthetic polymers with the antimicrobial efficacy of silver at the nanoscale [33]. These composite fibres can provide a controlled release of metal ions or reactive species, sustaining bactericidal concentrations over prolonged periods without the need for systemic administration.

Advanced electrospinning strategies further expand chemical control at the fibre level. Hydrogel‐forming systems produced via postspinning crosslinking maintain moisture at the wound interface, while coaxial electrospinning enables core–shell constructs that shield sensitive therapeutics and allow staged or asymmetric release profiles [34, 35]. A recent example by Terracina et al. demonstrates the potential of biodegradable PBS fibres loaded with ciprofloxacin (CPX) [28]. Their electrospun scaffolds showed uniform morphology, tunable fibre diameters based on CPX loading, membrane‐like wettability favourable for wound environments, and cytocompatibility above 90%. Importantly, CPX‐loaded fibres exhibited enhanced transdermal permeation and strong antibiofilm efficacy against *S. aureus* and *P. aeruginosa*, underscoring how electrospinning can modulate both delivery kinetics and antimicrobial performance.

### 2.5 Four‐Dimensional Printing of Responsive Materials

While 3D printing defines static architectures with controlled geometry and porosity, 4D printing introduces time as an additional design dimension, enabling printed materials to actively change shape, mechanical properties, or chemical behavior in response to external stimuli [36, 37]. In the context of antimicrobial and biomedical materials, this temporal programmability allows functionality to emerge, rather than being fixed at the moment of fabrication [38]. Among the available additive manufacturing techniques, FDM is currently the most widespread, cost‐effective, and exploitable 3D printing technology. It uses the layer‐by‐layer extrusion of thermoplastic filaments guided by computer‐aided design data [39]. In 4D printing applications, the use of FDM involves the processing of shape memory polymers (SMPs). These smart materials can undergo deformation and subsequently return to their original shape following the application of an appropriate stimulus, typically temperature [39]. However, the range of commercially available FDM‐compatible SMPs remains limited, and current research has primarily focused on poly(lactic acid) (PLA)‐ and polyurethane (PU)‐ based SMPs [40, 41].

Beyond shape‐memory behavior, 4D printing enables the fabrication of stimuli‐responsive biomaterials whose chemical and physical properties evolve over time, offering clear advantages for biomedical and antimicrobial applications. Unlike conventional 3D‐printed structures, which remain static after production, 4D‐printed materials can stiffen, degrade, self‐repair, or release bioactive molecules in a controlled manner, thereby mimicking the behavior of biological tissues, allowing controlled activation, release, or degradation to be programmed into the printed architecture [42, 43]. Smart biomaterials, including SMPs, stimuli‐responsive polymers (SRPs), and programmable hydrogels, are the key players in 4D printing. SRPs undergo reversible changes in shape or properties when exposed to specific triggers such as pH, temperature, light, electric, or magnetic fields [44]. These responses can be leveraged to trigger localized release of antimicrobial or bioactive agents in infection‐associated microenvironments, where pH often deviates from healthy tissue values. Similarly, photosensitive polymers enable spatially and temporally resolved activation, allowing drug release or antimicrobial action to be initiated on demand through light irradiation. For example, pH‐responsive polymers, such as polyacrylic acid (PAA), polymethacrylic acid, alginate, and chitosan, exploit ionizable functional groups to induce swelling, deswelling, or solubility changes in response to physiological pH changes [45].

Photosensitive polymers, including azobenzene‐based systems, spiropyran‐containing polymers, methacryloyl gelatin, and nitrobenzyl‐functionalized materials, enable spatially and temporally controlled drug release upon light irradiation [46, 47, 48]. Temperature‐sensitive materials represent another important class, in which temperature changes induce sol–gel transitions or volumetric changes. Examples include blends of PCL with poly(ethylene‐co‐octene), polyolefin elastomers, and PAA‐based systems [49]. Overall, these materials provide a versatile platform for the design of evolving, stimuli‐responsive architectures, positioning 4D printing as a powerful strategy for next‐generation adaptive biomedical devices.

Another field of application of these systems is wound management. Hydrogel‐based systems have an excellent adaptability capacity, as they are able to incorporate different active molecules, such as anti‐inflammatories, antibiotics, etc [50, 51]. Examples are represented by LM hybrid hydrogels. Eutectic gallium indium (EGaIn) is a very interesting LM [52]. Compared to traditional block hydrogels, 3D printing enables the fabrication of flexible, shape‐adaptive devices with spatially programmed responses, enhancing contact with irregular wound surfaces and improving therapeutic efficacy. Li et al. [53] have recently demonstrated the fabrication of a hybrid hydrogel fabricated by combining gelatin and gellan gum, with photothermal capacity, where light‐induced heating is used to generate localized antibacterial effects while simultaneously promoting tissue regeneration through the controlled release of growth factors such as VEGF. Beyond 4D printing, emerging fabrication paradigms such as so‐called “5D printing” are beginning to attract attention in biomedical materials science. In this context, 5D printing refers to multiaxis additive manufacturing approaches in which, in addition to the conventional three translational degrees of freedom, rotational axes are introduced, enabling the deposition of materials along curved and nonplanar trajectories [54]. This capability allows the fabrication of architectures that more closely mimic the anisotropy and hierarchical organization of biological tissues. From a chemical perspective, the introduction of multiaxis control expands the design space for confinement and microenvironmental engineering, as it enables the creation of continuous, curvature‐driven gradients in composition, porosity, and surface functionality [55]. Such features are particularly relevant for antimicrobial applications, where spatially resolved control of transport pathways and interfacial reactivity can be exploited to modulate bacterial adhesion and inactivation. Looking forward, 5D printing may open new opportunities for the fabrication of complex antibacterial micro‐ and nanorobotic systems, in which structural programmability, responsiveness, and localized chemical activity are integrated within a single platform. Although still at an early stage, this approach represents a promising extension of additive manufacturing strategies toward highly biomimetic and adaptive antimicrobial systems.

### 2.6 Other Printing and Coating Methods

In addition to high‐resolution and architecturally complex fabrication strategies, a range of solution‐based printing and coating methods remain highly relevant for antimicrobial material production, particularly where scalability, throughput, or thick‐film deposition are required. Screen printing and gravure printing provide alternative solution‐based approaches suitable for higher throughput and thicker film deposition. Screen printing, in particular, has been extensively employed for the fabrication of thick functional films, including gas sensors and catalytic or antimicrobial coatings. In this technique, paste‐like inks with high solids loading are forced through a mesh screen to create patterns with thicknesses ranging from a few micrometers to hundreds of micrometers. The rheological properties of screen printing pastes, including shear‐thinning behavior and thixotropy, are critical to achieving uniform coating and high resolution [56, 57].

Spray coating and spin coating, while less commonly associated with patterning, remain important techniques for depositing uniform metal oxide films over large areas, which can subsequently be patterned through photolithographic or other subtractive processes [58]. Spray coating offers advantages in terms of material utilization efficiency and the ability to coat nonplanar or textured substrates, while spin coating provides excellent thickness uniformity on flat substrates [59]. An emerging approach to metal oxide printing exploits LMs as dynamic precursors for oxide film formation. In this strategy, LM droplets or films spontaneously form a thin oxide layer at their surface upon exposure to oxygen‐containing atmospheres. This native oxide skin can reach nanometer‐scale thicknesses and exhibits mechanical properties that allow it to be peeled, transferred, or manipulated as a free‐standing membrane.

The LM‐mediated approach offers several distinctive advantages for oxide printing. First, the oxide layer forms spontaneously and conformally, adapting to the shape of the underlying LM. Second, the liquid state of the precursor enables dynamic reconfigurability and self‐healing properties. Third, the native oxide can be repeatedly harvested and reformed, providing a potentially sustainable route to oxide film production. The oxide layers can be transferred onto diverse substrates, including flexible polymers, textured surfaces, and temperature‐sensitive materials that would be incompatible with conventional high‐temperature oxide deposition.

Pattern formation with LM‐mediated oxides can be achieved through several routes. Stamping or contact printing techniques allow transfer of oxide films in defined patterns by bringing the LM into contact with a substrate in selected regions. Stencil masks can be employed to confine LM spreading and thereby pattern the resulting oxide. More recently, oxide structures have been generated through controlled oxidation of LM patterns created by injection into microfluidic channels, printing through nozzles, or deposition onto patterned substrates. The properties of LM‐mediated oxides depend strongly on the oxidation conditions and subsequent processing. Controlled thermal annealing in oxygen‐rich atmospheres can drive crystallization, improve stoichiometry, and modify the electronic properties of the oxide. However, the relatively low processing temperatures achievable with this approach may limit crystallinity compared to conventional high‐temperature synthesis routes, presenting trade‐offs between substrate compatibility and material quality [60, 61]*.*

## 3 Chemistry of Materials for Tailored Antimicrobial Functionality

### 3.1 Polymers Characteristics

**FIGURE 2:**
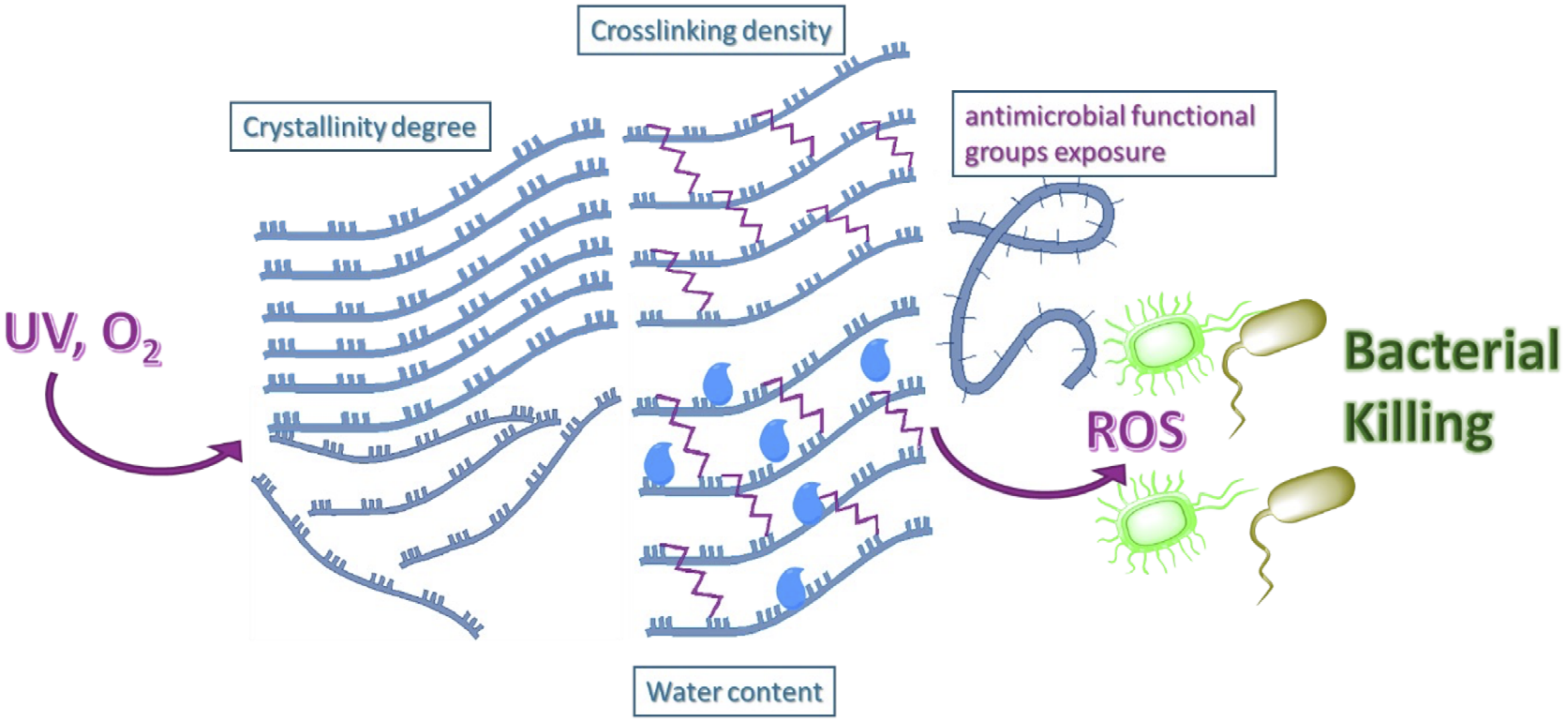
Structural parameters of the polymer matrix regulate the diffusion of oxygen and reactive oxygen species (ROS) within the network, thereby influencing the antibacterial activity.

In the landscape of advanced antimicrobial surfaces, polymeric materials represent an exceptionally versatile platform, capable of combining chemical functionality, structural control, and mechanical adaptability [62]. Nevertheless, the antimicrobial performance of such systems cannot be assessed solely in terms of initial activity but must be evaluated in relation to the long‐term structural and chemical stability of the polymer matrix [63]. In this context, it has been widely demonstrated that the formation of radical species can simultaneously contribute to bacterial inactivation [64]. In thermoplastic polymers and thermosetting resins, structural parameters such as crystallinity degree, molecular weight, and crosslinking density govern the diffusion of oxygen and reactive species within the matrix [65]. Radical formation induced by UV irradiation, mechanical stress, or photocatalytic processes can therefore enhance antimicrobial activity at the expense of progressive chain scission and loss of mechanical and functional stability (Figure 2). Hydrogel networks introduce a distinct paradigm, based on highly hydrated and dynamically responsive matrices. In these systems, swelling degree and crosslinking density regulate chain mobility and the exposure of antimicrobial functional groups [66]. The high‐water content enhances interactions with bacterial membranes and improves the efficacy of labile antimicrobial moieties but simultaneously accelerates radical diffusion and hydrolytic degradation of the polymer matrix [67]. As a result, antimicrobial stability in hydrogels arises from a delicate balance between permeability, structural flexibility, and resistance to degradation under prolonged oxidative stress conditions [68]. Nanofibrous matrices, typically produced by electrospinning, combine a high surface‐to‐volume ratio with a surface topography intrinsically hostile to bacterial adhesion [69]. The extensive surface exposure renders nanofibrous systems particularly susceptible to radical formation and photo‐oxidative degradation of the polymer matrix, with a direct impact on the durability of antimicrobial activity [70]. The loss of antimicrobial efficacy is frequently associated with matrix reorganization or degradation, which alters the orientation and surface density of active sites, thereby diminishing interfacial impact on the pathogen [71]. Fine control over structural parameters ranging from crystallinity and crosslinking density to nanofibrous morphology and hydrogel network dynamics thus emerges as a fundamental strategy to confine radical activity at the bacteria–material interface, minimize matrix degradation, and ensure sustained antimicrobial performance over time [72].

### 3.2 Metals and Metal Oxides

The solid‐state chemical speciation of printed metal oxides is a critical factor that dictates electronic properties, chemical stability, and biological (antimicrobial) activity [73]. Unlike bulk materials, printed films exhibit an extremely complex surface chemistry due to incomplete sintering processes and the presence of organic residues originating from ink solvents and surfactants. The chemical speciation of printed metal oxides is strongly influenced by the precursor chemistry and printing methodology.

When printing metal oxides via inkjet, sol–gel, or other solution‐based routes, the material transitions from liquid precursors (often nitrates, acetates, or chlorides) to a solid network. The final speciation is rarely 100% pure oxide but rather an equilibrium between different phases: crystalline bulk material (e.g., hexagonal wurtzite ZnO), amorphous or disordered phases at interfaces between particles where cross‐linking is incomplete, mixed oxidation states (particularly relevant for copper with Cu^0^, Cu_2_O, CuO and titanium with Ti^3+^, Ti^4+^), and surface functional groups including hydroxyls (−OH), carbonates (CO_3_^2‐^), and carbonaceous residues that influence zeta potential and interactions with bacterial membranes [74].

Analytical techniques for characterizing solid‐state speciation include X‐ray photoelectron spectroscopy (XPS) for surface oxidation states and the ratio of lattice oxygen to adsorbed oxygen (oxygen vacancies), X‐ray diffraction (XRD) for identifying crystalline phases (e.g., distinguishing between tenorite CuO and cuprite Cu_2_O), X‐ray absorption spectroscopy (XAS/EXAFS) for local speciation around metal atoms even in ultra‐thin or amorphous films, and Fourier‐transform infrared spectroscopy and Raman spectroscopy for identifying specific metal–oxygen bonds and residues of organic ink ligands [75, 76, 77].

Speciation directly governs ion release kinetics, which drive biocidal activity. Oxygen vacancies (*V*_O_) in printed oxides such as TiO_2_ or ZnO act as active sites for reactive oxygen species (ROS) generation [78]; speciation rich in surface defects drastically increases antimicrobial efficacy. Phase solubility differences are significant: for printed copper, Cu_2_O (copper I oxide) is often more effective than CuO (copper II oxide) due to faster release of Cu^+^ ions, which exhibit higher toxicity to microorganisms. Surface hydration and the formation of hydroxylated layers (M‐OH) determine how materials interact with bacterial biofilms and cellular interfaces [79].

Defect engineering through controlled speciation represents a powerful tool for tailoring biomedical performance. Printing parameters, including annealing temperature, atmosphere composition (oxidizing, reducing, or inert), and heating rate, can be manipulated to control oxygen vacancy concentration, phase composition, and surface chemistry. This recognition that printing is not merely a deposition method but rather a tool to control solid‐state chemistry opens new avenues for rational design of biomedical metal oxides with optimized antimicrobial potency and biocompatibility profiles [80, 81].

Solution‐based techniques, including inkjet, aerosol jet, screen, and gravure printing, commonly employ metal salts, metal–organic complexes, or metal–organic decomposition inks [82, 83]. These approaches often result in precursor‐derived amorphous networks, where residual ligands, hydroxyl groups, and mixed metal oxidation states persist prior to complete oxide formation [84]. Consequently, printed films frequently exhibit nonstoichiometric compositions and a high density of intrinsic defects, such as oxygen vacancies and metal interstitials, which strongly affect charge transport and surface reactivity [85].

Understanding solid‐state chemical speciation in printed oxides has been greatly advanced by modern spectroscopic techniques. XPS is widely used to probe surface‐sensitive oxidation states and defect‐related states. XAS, including XANES and EXAFS, provides element‐specific information on local coordination environments and short‐range order, even in amorphous [86]. Complementary techniques such as Raman spectroscopy and solid‐state NMR have proven essential for identifying residual precursor species and amorphous oxide networks that are undetectable by conventional XRD [87, 88, 89]. Recent studies demonstrate that controlling chemical speciation is not merely a processing challenge but a powerful materials design strategy. Intentional stabilization of metastable phases, tuning of defect populations, and control over mixed oxidation states have enabled improved performance in oxide thin‐film transistors, gas sensors, and neuromorphic devices [90, 91]. However, reproducibility remains a significant challenge due to the complex interplay between ink formulation, printing dynamics, and thermal history. In conclusion, the solid‐state chemical speciation of printed metal oxides is a decisive factor governing their functional properties. Future progress in this field will require integrated strategies combining rational precursor design, low‐temperature processing, and in situ or operando characterization methods. Such approaches are essential to establish robust structure–property relationships and to enable the scalable manufacturing of high‐performance printed oxide‐based devices.

### 3.3 Antimicrobials Encapsulated in Biomaterials

Recent advances in biomaterials engineering have enabled the development of sophisticated platforms for the encapsulation, immobilization, and controlled release of antimicrobial agents, with the dual objective of enhancing antimicrobial efficacy and mitigating the emergence of AMR. In this context, several strategies have been explored, ranging from surface functionalization with AMPs to microencapsulation techniques and advanced manufacturing approaches such as electrospinning and 3D printing. The following section critically reviews representative studies that exemplify these approaches, focusing on their design principles, fabrication methodologies, and antimicrobial performance.

**FIGURE 3:**
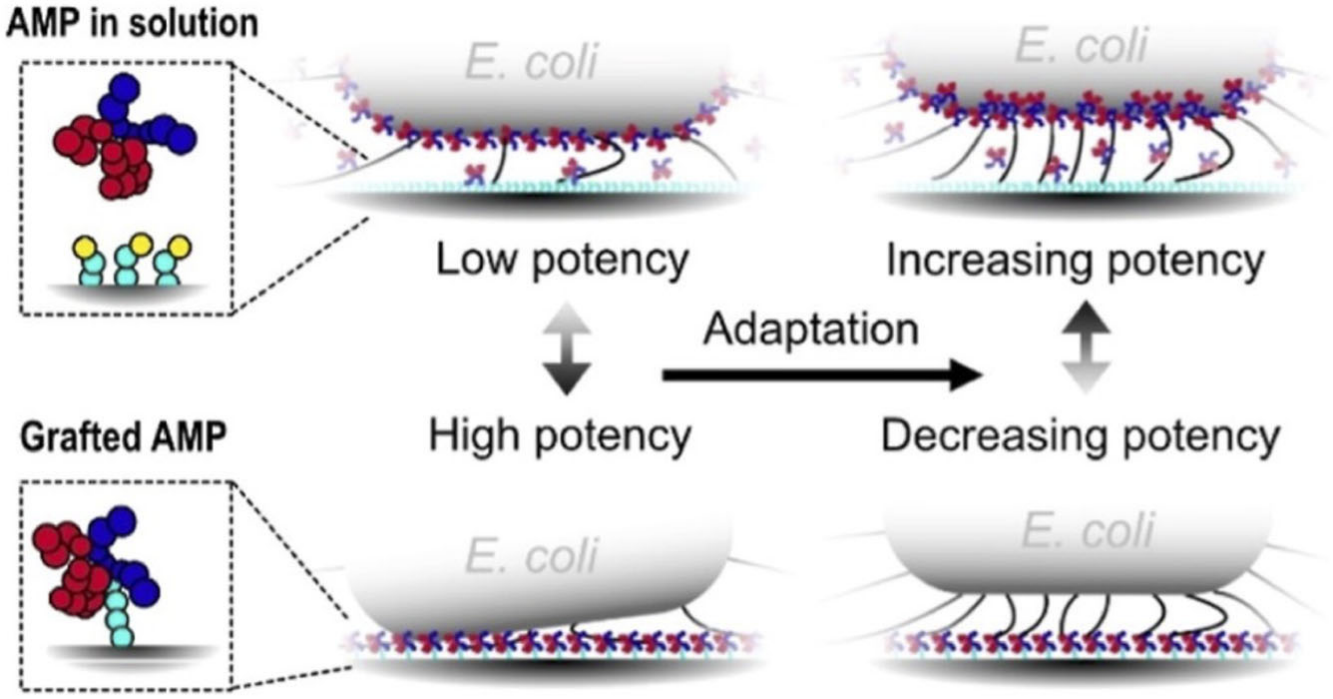
Surface coatings with AMP in preventing formation of biofilm. Adapted from ACS Applied Materials & Interfaces, ref. [92] Copyright 2024. This is an open access article distributed under the terms of the Creative Commons CC BY 4.0 license.

A notable example of surface‐based antimicrobial biomaterials is provided by Hansson et al. [92], who developed polymeric coatings functionalized with covalently grafted AMPs to prevent bacterial adhesion and biofilm formation by *E. coli*. The peptides were immobilized onto solid substrates using click chemistry, which allowed precise control over peptide density and ensured stable anchoring to the surface (Figure 3). Through a combination of live‐cell microscopy, microfluidic systems, and automated image analysis, the authors systematically investigated how surface‐bound AMPs affect bacterial growth, morphology, and attachment dynamics. Their experiments demonstrated that grafted AMPs initially inhibited bacterial proliferation more efficiently than their soluble counterparts. However, prolonged exposure led to adaptive changes in bacterial adhesion mechanisms, particularly through increased expression of type‐1 fimbriae, which altered the bacterial binding mode and reduced the antimicrobial efficacy of the coating. This work provides mechanistic insights into how immobilized antimicrobial agents interact with bacterial cells and highlights the importance of understanding bacterial adaptive responses when designing long‐term antimicrobial surfaces.

An alternative and highly practical approach is described by Ocepek et al. [93], who developed antimicrobial cotton fabrics through the deposition of triclosan‐loaded polymeric microcapsules. In this study, triclosan was encapsulated within melamine‐formaldehyde polymer shells using an in situ polymerization process, producing microcapsules with diameters primarily in the 4–8 μm range. These microcapsules were then incorporated into a printing paste and applied onto cotton fabrics via flat‐screen printing, followed by drying and thermal curing. The antimicrobial activity of the treated textiles was evaluated against both *S. aureus* and *E. coli*. The results showed that fabrics printed with higher microcapsule loadings (100 g/kg of printing paste) exhibited excellent antibacterial activity, producing large inhibition zones against both bacterial strains, even after repeated washing cycles. Scanning electron microscopy revealed a homogeneous distribution of intact microcapsules on the fiber surface, confirming the mechanical stability of the coating. Furthermore, washing significantly reduced free formaldehyde content, improving the safety profile of the material. This study clearly demonstrates how microencapsulation combined with scalable textile printing technologies enables durable and controlled antimicrobial performance, particularly suitable for technical and healthcare textile applications.

### 3.4 Dual‐Function Antimicrobial Materials

The same chemical and structural design principles that govern antimicrobial activity can be extended to impart diagnostic functionality, enabling materials that simultaneously detect, monitor, and treat bacterial infections. In this context, antimicrobial materials evolve into theranostic platforms, where therapeutic action and sensing capabilities are intrinsically integrated within a single engineered construct. Rather than relying on separate diagnostic and treatment steps, these systems exploit tailored material chemistry to couple biological recognition with localized antimicrobial response [94, 95].

**FIGURE 4:**
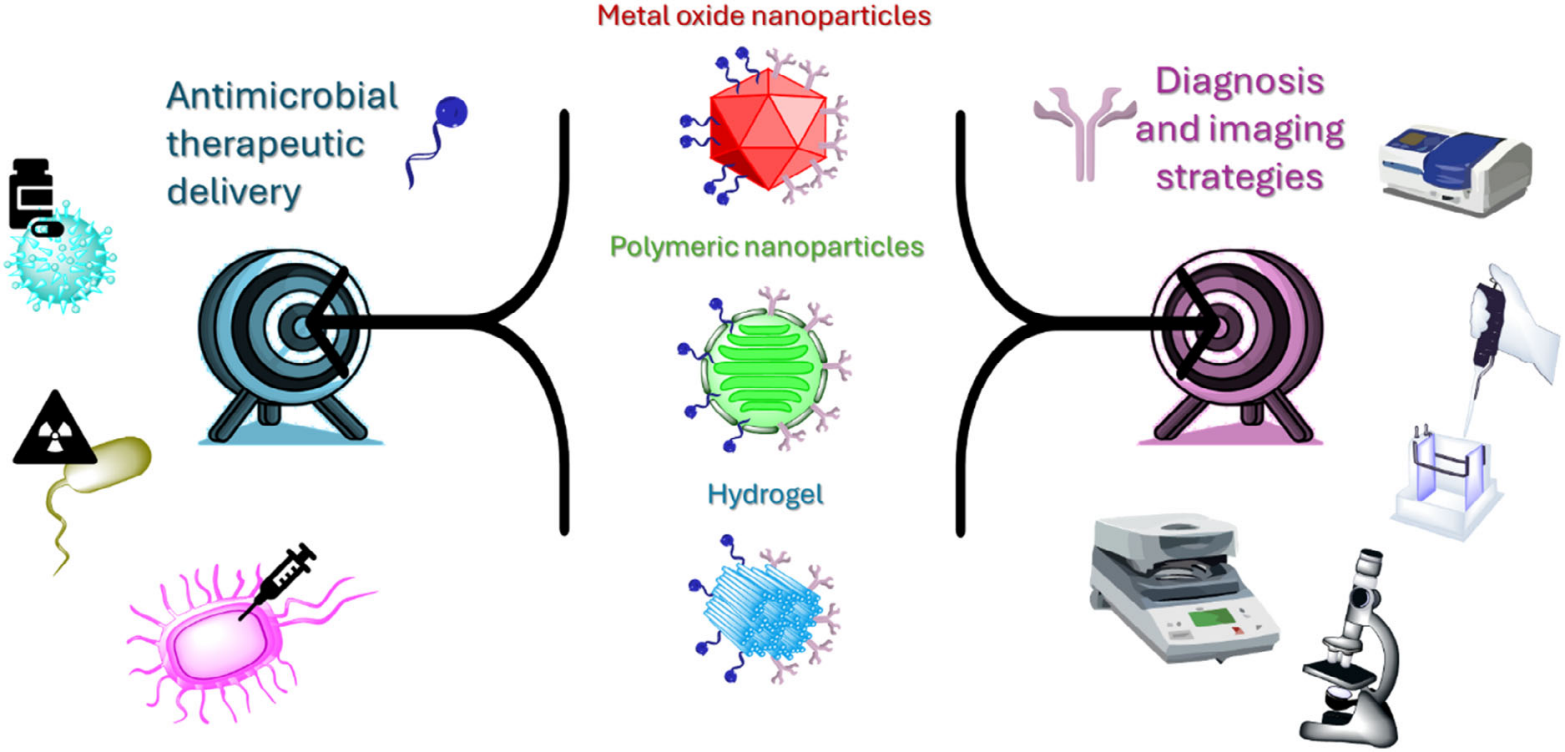
Schematic representation of nanoparticle‐based and hydrogel platforms exploited for dual therapeutic and diagnostic applications in the context of bacterial infection management.

At the core of theranostics lies the ability of materials to transduce biological events into measurable physical or chemical signals. This is commonly achieved through the incorporation of imaging agents, fluorescent probes, redox‐active components, or responsive reporters within antimicrobial matrices [96]. When bacteria adhere, proliferate, or form biofilms, changes in local pH, enzymatic activity, or metabolite concentration can be detected by responsive materials, providing real‐time information on infection onset and progression that is coupled to the therapeutic effect. One notable strategy involves the design of nanodrugs based on ciprofloxacin derivatives that display both enhanced antibacterial activity and intrinsic luminescence for bacterial imaging. In this approach, molecular modifications of the antibiotic produce nanoaggregates with aggregation‐induced emission properties, enabling them to act as theranostic agents capable of imaging and killing resistant *E. coli* strains while lowering minimum inhibitory concentrations (MICs) compared to the parent drug. The enhanced activity of ciprofloxacin observed in this study, together with the reduction in MIC values, indicates that lower antibiotic doses may achieve effective antimicrobial activity and improved outcomes. From an AMR perspective, achieving efficacy at lower concentrations is particularly important, as it may reduce the selective pressure associated with higher antibiotic exposure and may support the therapeutic administration of lower antibiotic doses. This approach represents a modular strategy for designing pure theranostic nanodrugs that are both therapeutic and detectable in situ, providing a tool for tracking infection and treatment efficacy simultaneously. In another example, stimuli‐responsive hydrogels have been developed that change their optical or fluorescent output in response to bacterial infection while also delivering antimicrobial action, specifically in the context of wound healing. These hydrogels incorporate elements such as AMPs and polydopamine nanoparticles that produce visible signals in response to infection‐triggered biochemical changes. By linking imaging contrast with on‐demand antibacterial functionality, these materials provide visual feedback on infection status and can guide targeted treatment [94, 95]. Nanostructured and composite materials are particularly well suited for theranostic applications because their high surface area and tunable chemistry facilitate both interaction with bacteria and signal transduction. Metal and metal oxide nanomaterials, for example, can simultaneously generate ROS for bacterial inactivation and act as contrast agents for optical or electrochemical sensing. Similarly, polymeric nanocarriers and hydrogels can be functionalized with fluorophores or imaging moieties while encapsulating antimicrobial agents, allowing infection‐responsive release to be correlated with diagnostic readouts (Figure 4).

Targeting specificity is a key advantage of theranostic antimicrobial materials. By exploiting bacterial surface markers, biofilm‐associated components, or enzymatically cleavable motifs, materials can be engineered to preferentially interact with microbial cells rather than host tissue. This selectivity enhances diagnostic accuracy while minimizing off‐target toxicity. In biofilm‐targeting systems, theranostic nanocarriers that accumulate within the extracellular polymeric matrix enable both visualization of biofilm formation and localized disruption through chemical or photochemical mechanisms. The integration of imaging and therapy is particularly relevant for chronic and implant‐associated infections, where early‐stage biofilm formation is difficult to detect, and conventional treatments are often ineffective.

## 4 Confinement and Microenvironmental Control

### 4.1 Nanoscale Confinement

**FIGURE 5:**
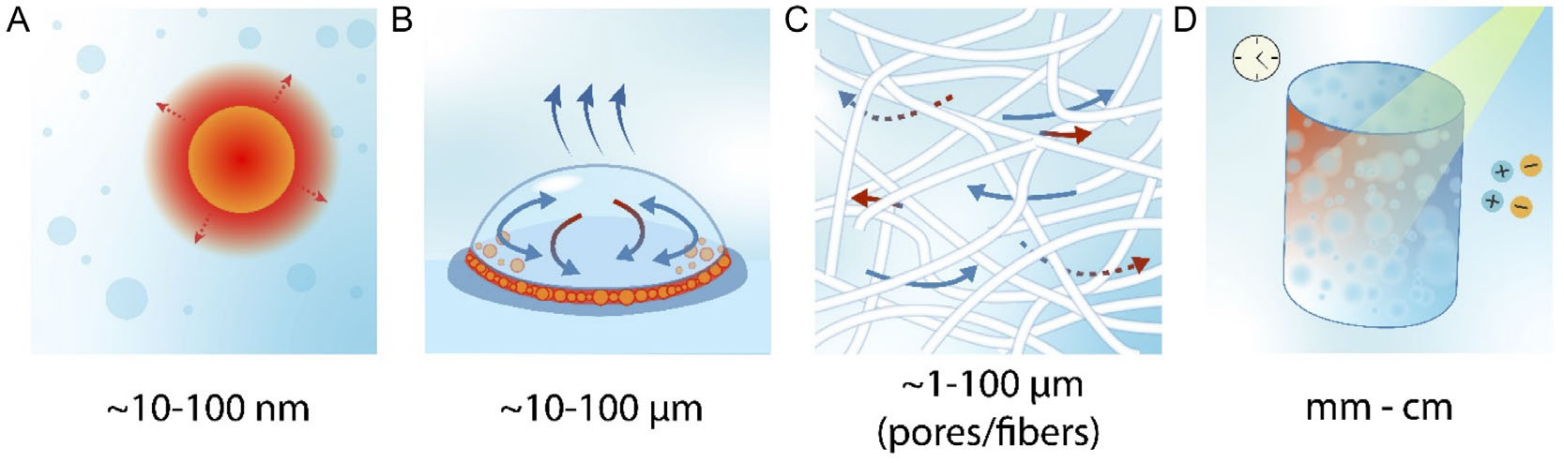
Confinement and microenvironmental control across increasing length scales in antimicrobial materials. From nanoscale domains (~10–100 nm), where interfacial energy and restricted diffusion govern local chemical activity (A), to micrometer‐sized compartmentalized droplets (~10–100 µm) sustaining reaction‐diffusion processes (B), and interconnected fibrous architectures (~1–100 µm pores/fibres) that impose diffusion‐limited transport along high‐area interfaces (C), and confinement progressively extends to multiscale 3D/4D printed constructs (mm‐cm) (D).

Nanoscale confinement represents a transformative paradigm in the chemical design of antimicrobial platforms, moving beyond the simple delivery of molecular agents to the fabrication of engineered physicochemical microenvironments with potentiality in innovative antimicrobial strategies [97] (Figure 5).

This approach can be pursued, for example, by combining printing techniques with the phenomenon of liquid–liquid phase separation [97, 98]. The formation of complex condensates and artificial membranelles organelles leads to molecularly crowded microdomains that can be arranged in a spatial functional fashion, taken over by the printing approach. The physical chemistry of these condensates is governed by an ultralow interfacial tension, typically between 0.01 and 0.5 mN/m, and a relatively broad interfacial width ranging from 8 to 20 nm, which allows for superior molecular mobility and flexibility compared to rigid lipid membranes [99]. The combination of ultralow interfacial tension and broad interfacial width makes these condensates attractive for the controlled and sustained release of antimicrobial molecules, enabling precise spatial and temporal delivery that would be difficult to achieve with rigid membrane‐based systems. This unique interfacial architecture is driven primarily by the massive entropic gain resulting from the release of small counterions when oppositely charged polyelectrolytes associate, a principle that has been leveraged to assemble high‐potency antimicrobial nanoparticles [100].

**FIGURE 6:**
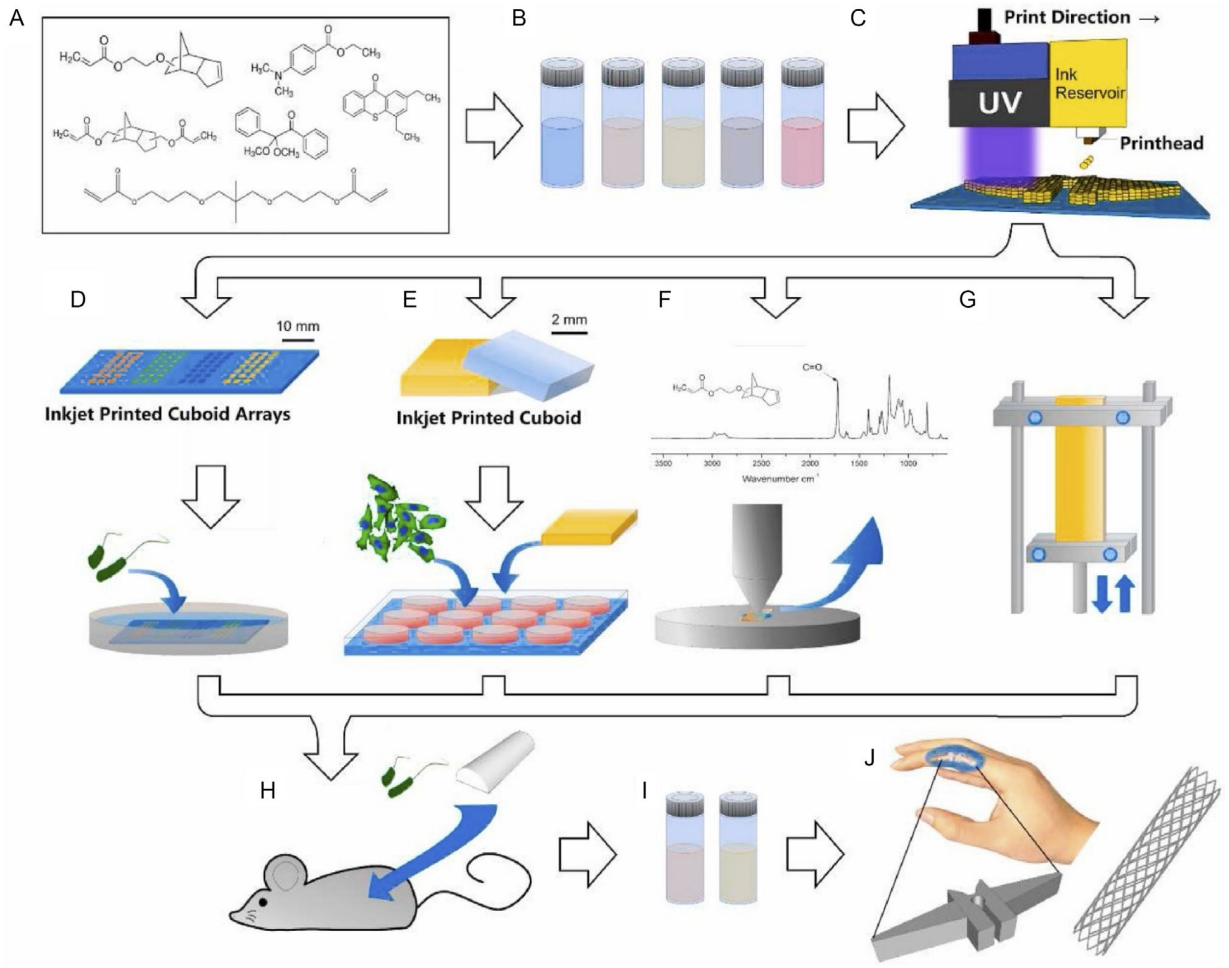
Schematic for developing optimized formulations for ink‐jet based 3D‐printing. (A and B) Monomer candidates selected for formulation development and optimization. (C) A Fujifilm Dimatix DMP‐2830 printer was used to print samples. The system in this case was equipped with a cartridge ejecting 10 pL drop volumes, utilizing up to 16 nozzles. (D) On‐slide arrays of cuboids were created by ink‐jet based 3D‐printing for preliminary microbiology biofilm assays using *P. aeruginosa*. (E) Cytotoxicity and cell attachment biocompatibility tests on the printed samples were carried out using mouse embryonic fibroblast 3T3 cells to assess biocompatibility of the printed device; (F) attenuation total reflectance infrared spectroscopy was used to quantify the levels of residual acrylate in the specimens made from different ink formulations; (G) mechanical tests were performed by dynamic mechanical analysis in tension mode at room temperature; (H) formulations resulting in desirable properties were tested in vivo to ensure that the cell instructive properties were retained in a more complex environment; and (I and J) the finalized ink formulations were used to print concept devices. Adapted from Biomaterials, ref. [9] Copyright 2022. This is an open access article distributed under the terms of the Creative Commons CC BY 4.0 license.

First, several examples of effective condensate‐based antimicrobial systems can be reported. For instance, the supramolecular assembly of colistin into 8 nm nanoparticles through coacervation with polyanionic peptides has demonstrated an improved safety profile and reduced nephrotoxicity while maintaining equivalent or superior bactericidal efficacy against multidrug‐resistant Gram‐negative pathogens such as *K. pneumoniae* and *A. baumannii* [100]. Recent advances in the stabilization of these confined domains have introduced universal membrane‐forming agents based on block polymers with “*condenophilic*” (that likes the condensed phase) and “*condenophobic*” (that prefers the dilute phase) segments, which utilize multiple modes of interaction, including amidoamine‐carboxylate electrostatic attraction and cation–π coordination, to target diverse chemical flavors of condensates, ensuring their structural integrity even under harsh environmental conditions [101]. The engineering of patterned domains and the exploitation of charge anisotropy on protein surfaces have further refined the selective uptake of antimicrobial cargo. For example, the presence of specific cationic patches on lysozyme enables its preferential partitioning into coacervate phases even at pH levels where its net charge might suggest otherwise, facilitating the design of highly loaded biocatalytic crucibles [98]. In the realm of sustained disinfection, putty‐like complex coacervates based on poly(allylamine hydrochloride) and tripolyphosphate have proven capable of encapsulating hydrophobic biocides like triclosan, extending its release over multiple months due to the high network density within the confined domain [102]^.^ Similarly, silver‐doped phosphate coacervate gels provide a resorbable platform for the continuous release of Ag^+^ ions, achieving significant log reductions in bacteria associated with chronic wound infections while supporting keratinocyte viability [103]. A particularly exciting frontier in this field involves the use of advanced printing technologies to fabricate or spatially arrange confined microenvironments with unprecedented precision and scalability. Recent work has demonstrated that inkjet printing can be employed to develop from bioadhesive arrays to stable surfactant‐stabilized aqueous compartments ranging from the nanoliter to the femtoliter scale [104, 105, 106]. These droplets, stabilized by oil confinement and mild surfactants like Tween‐20, function as specialized compartments where molecular interactions can be monitored at nanomolar concentrations using advanced techniques like raster image correlation spectroscopy. By moving from picoliter to femtoliter‐scale droplets, achieved through the design of high‐precision “spike waveforms” that minimize ejection time, the same research group has uncovered the emergence of autonomous molecularly crowded structures [107]. In these few‐micrometer regimes, surface energy minimization leads to the spontaneous formation of thin‐shell molecular annuli where molecules are highly up‐concentrated. These fL‐droplets can host complex biochemical reactions, such as mitochondrial‐like enzymatic processes (CYP2E1) or DNA‐hairpin sensors, showing that confinement at this scale induces spatial heterogeneity and alters molecular responsivity to external triggers. The combination of molecular condensates and advanced printing technologies represents a relatively under‐explored yet highly promising research frontier. A recent study demonstrates the application of 3D inkjet printing to fabricate a multifunctional bilayer scaffold that integrates apoptotic extracellular vesicles and antibacterial coacervates to promote wound healing and prevent infection [9] (Figure 6).

The scaffold's lower layer, featuring large pores, continuously releases apoptotic vesicles to stimulate angiogenesis and collagen deposition, while the upper layer, with dense pores, releases pH‐responsive curcumin‐containing coacervates to provide antibacterial activity. In vivo, this scaffold accelerated wound closure, enhanced collagen organization, and significantly enhanced intrinsic bacterial resistance. Integrating these inkjet‐based approaches offers immense potential for fabricating next‐generation antimicrobial systems. In addition, since inkjet printing allows for the simultaneous, multichannel dispensing of different bio‐inks, it is possible to program “cellular‐like” microreactors that host multiple antimicrobial agents or enzymatic cascades within individual, addressable droplets. This could lead to the development of biochips for high‐throughput screening of antimicrobial efficacy or the creation of smart coatings that release agents only upon sensing specific microbial triggers.

### 4.2 Compartmentalized Droplet Microreactors

Microfluidic technology has emerged as a highly promising platform for the design and implementation of microreactors and microstructured systems in a precisely controlled way [108, 109]. By leveraging the inherent advantages of microscale architectures, such as high surface‐to‐volume ratios, programmable fluid flow, and enhanced heat and mass transfer, these systems allow for the strict regulation of reaction parameters and the engineering of specific molecular environments with unprecedented accuracy [110]. The implementation of droplet microfluidics introduces a superior level of control in the engineering of antimicrobial platforms, effectively transforming discrete volumetric fractions into isolated, transient compartments that function as individual microreactors [111]. In these systems, the compartmentalization of reagents into pico‐ and nanoliter volumes allows for the confinement of chemical and biological species, significantly accelerating mixing kinetics through chaotic advection processes triggered as droplets navigate through winding microchannels [112]. This phenomenon drastically reduces diffusion distances and ensures a uniform distribution of reactants, overcoming the inherent mass‐transfer limitations of bulk reactors where concentration and thermal gradients are often difficult to regulate. Reactivity within these microcompartments is profoundly influenced by molecular crowding effects, which closely mimic the dense, macromolecular environment of the cellular cytoplasm [113, 114]. Research has demonstrated that molecularly crowded microenvironments, such as those generated within coacervate microdroplets, can sequester and protect labile antimicrobial agents, including enzymes and peptides, from denaturation, thereby modulating their biochemical stability and prolonging their functional lifespan. Such architectures enable the formation of complex, multicompartmental systems, e.g., “droplet‐in‐droplet” or “onion‐like” coacervates, able to sustain spatially regulated reaction–diffusion processes [113]. This architectural precision is vital for the development of “smart” strategies against AMR, where the release of active biocides or the activation of enzymatic cascades can be synchronized with local environmental triggers or external physical stimuli. Furthermore, droplet‐based microreactors excel in generating stable and programmable concentration gradients, which are fundamental for high‐throughput antimicrobial susceptibility testing (AST) [109]. The stochastic isolation of individual bacterial cells within droplets of culture media allows for the real‐time monitoring of proliferation and drug response, significantly reducing the diagnostic window compared to conventional methods [111]. Advanced manipulation techniques, such as using alternating current electric fields to induce droplet oscillation or magnetic fields to actuate internal Fe‐based microspheres, have shown that active physical agitation within these compartments can dramatically enhance mass transfer and solid–liquid contact [115]. This not only improves the efficiency of Fenton‐like reactions for antibiotic degradation but also provides a dynamic environment for studying bacterial behavior under precise chemical stress. Finally, compartmentalization serves as a robust tool for the in situ synthesis of antimicrobial nanomaterials, such as AgNPs or heterogeneous bio‐photocatalysts [112]. By finely regulating residence times and precursor delivery within the droplets, it is possible to achieve nanoparticles with extremely narrow size distributions and superior long‐term stability. Integrated systems that combine micromixers with local heating and temperature sensors allow for the synthesis of photocatalysts, e.g., GOx, ZnO, and TiO_2_, that have already demonstrated their great potentiality for photocatalysis in biological systems [116, 117, 118], which leads to degradation of model organic molecules and antibiotics up to mineralization, as reported for amoxicillin with a reaction rate more than 40 times higher than bulk systems [110, 119]. This underscores how the chemical design of compartmentalized microreactors is key to maximizing therapeutic efficacy while minimizing the chemical load on the environment.

The chemical design of these compartmentalized systems gains further potential when microfluidic architectures are directly integrated with printing technologies, enabling the transition from static fabrication to programmable and dynamic reaction platforms. 3D printing enables the fabrication of microfluidic devices with high design flexibility and submicron resolution, facilitating the integration of multiple functional modules on a single chip for high‐throughput process intensification [120]. Within these platforms, droplet‐based microfluidics plays a pivotal role by transforming discrete fluid volumes into isolated, transient microreactors that provide precise spatiotemporal control over chemical and biological processes [121]. The adoption of active digital microfluidic techniques driven by magnetic or electric fields enables the automated manipulation of trace samples for applications ranging from rapid AST to the controlled synthesis of functional nanomaterials. For instance, ferro‐robotic systems inspired by automated logistics have been designed to manipulate biological droplets for rapid viral detection, such as COVID‐19 screening, through automated splitting, merging, and mixing on‐chip [122]. Furthermore, 3D‐printed modular platforms allow for the integration of sensors and microvalves within a single device, facilitating the high‐throughput screening of personalized antimicrobial therapies [120].

### 4.3 Fibrous Nano‐Architectures

Fibrous nano‐architectures represent a distinct mode of antimicrobial confinement where chemical control is exerted through continuous and highly interconnected interfacial networks rather than isolated microdomains. Electrospun nanofibrous membranes exhibit a very high surface‐to‐volume ratio and porosity, which enhances interactions with microbes and facilitates localized chemical activity [69]. In composite and functionalized electrospun fibres, antimicrobial agents can be incorporated directly into the polymer matrix or immobilized on the fibre surface [28, 123, 124]. This approach allows the simultaneous presentation of antimicrobial chemistry and structural confinement, enabling sustained release or contact‐dependent activity. Electrospun nanofibers with embedded biocidal nanoparticles such as silver or copper show enhanced antibacterial performance against common pathogens due to the high interfacial area between fibres and bacteria [26]. The structural features of electrospun nanofibers, including fine diameters and pore interconnectivity, establish diffusion‐limited pathways that regulate the transport of antimicrobial agents and other bioactive molecules, making these systems effective for sustained antimicrobial action [125]. Moreover, specific experimental studies have demonstrated the feasibility of metal‐doped electrospun nanofibrous membranes with broad antimicrobial activity. For example, cellulose acetate electrospun mats incorporating metal‐doped silica nanoparticles (Ag, Cu, and Co) exhibited self‐sterilizing behavior and significant inhibition of bacterial and fungal strains, showing how fibre composition and embedded nanoparticles affect antibacterial activity [126]. Electrospun nanofibers also offer versatile platforms for multimodal functionality. When used in wound dressings and regenerative scaffolds, functionalized biopolymer nanofibers can support cell growth while delivering antimicrobial and other therapeutic agents, mimicking the extracellular matrix and enabling localized therapy with minimal systemic exposure [127].

### 4.4 Multiscale Constructs in 3D/4D Materials

In 3D‐ and 4D‐printed constructs, confinement and microenvironmental control extend beyond the local scale to become spatially and temporally organized across the entire architecture. Unlike nanoscale patterning or fibrous systems, where confinement primarily emerges from local geometry and interfacial effects, 3D‐ and 4D‐printed materials enable the deliberate programming of chemical heterogeneity over millimeter‐ to centimeterscale constructs. This capability allows gradients in composition, porosity, stiffness, hydration, and transport properties to be embedded directly into the material during fabrication, generating hierarchically structured antimicrobial microenvironments rather than isolated active domains.

In such multiscale architectures, bacterial interaction with the material is inherently dynamic and sequential. As microorganisms migrate, adhere, and potentially proliferate within or on the surface of a printed construct, they encounter a succession of chemically distinct microenvironments characterized by different diffusion regimes, local concentrations of antimicrobial agents, and mechanical constraints. Spatial modulation of pore size and interconnectivity governs molecular transport and ion diffusion, while gradients in polymer chemistry or filler distribution regulate the availability and lifetime of reactive species. As a result, antimicrobial activity arises from the coordinated action of structure and chemistry across multiple length scales, rather than from a single localized mechanism [128, 129].

3D printing technologies provide precise control over these architectural parameters. By tuning infill geometry, filament orientation, and layer stacking, it is possible to engineer diffusion‐limited regions that sustain elevated local concentrations of metal ions or ROS while minimizing systemic exposure. This approach is particularly relevant for composite systems containing metal or metal oxide nanoparticles, where spatial confinement within the polymer matrix directly influences ion release kinetics, ROS generation, and long‐term antimicrobial efficacy [16, 17]. In this context, confinement acts not only as a physical barrier but also as a chemical regulator that shapes reaction‐diffusion processes within the scaffold.

The introduction of a temporal dimension in 4D printing further expands the concept of confinement by enabling microenvironments to evolve in response to external stimuli. Shape‐memory polymers, stimuli‐responsive hydrogels, and degradable networks allow antimicrobial architectures to change geometry, permeability, or chemical activity over time. These dynamic transformations can be triggered by temperature, pH, light, or moisture, conditions that are often altered in infected or inflamed tissues [37, 38]. In such systems, confinement is no longer static but emerges transiently, for example through swelling‐induced pore closure, stimulus‐triggered release of antimicrobial agents, or progressive degradation that exposes new reactive interfaces. This temporal programmability enables antimicrobial activity to be synchronized with environmental cues, reducing unnecessary exposure to biocidal agents and limiting the selection pressure associated with continuous low‐dose release. For instance, pH‐responsive or photothermally active hydrogels can remain chemically inert under physiological conditions but rapidly generate bactericidal microenvironments upon infection‐induced changes or external activation [43, 53, 130]. In this framework, antimicrobial efficacy is tightly coupled to the dynamic regulation of confinement, rather than to permanent material properties alone.

## 5 Antimicrobial Mechanisms in Tailored Constructs

In printed and microstructured antimicrobial materials, bactericidal activity emerges from the interaction between microbial cells and chemically confined microenvironments engineered through fabrication. In contrast to conventional dispersed systems, where antimicrobial mechanisms are governed by bulk concentrations and homogeneous exposure, printed constructs impose spatial and temporal constraints that fundamentally alter how bacteria experience chemical stress. As a result, mechanisms such as ROS generation, ion release, membrane disruption, and intracellular interference cannot be regarded as intrinsic properties of metals or metal oxides alone, but rather as architecture‐dependent responses shaped by confinement, defect chemistry, and interfacial design [131].

Printing technologies introduce control over surface morphology, phase composition, defect density, porosity, and transport pathways, enabling antimicrobial action to be localized at the bacteria–material interface. This localization allows high local reactivity to be achieved while minimizing total material load and uncontrolled leaching, a key advantage in the context of AMR. From a mechanistic standpoint, the antimicrobial activity of these systems can be broadly classified into four main categories: (i) oxidative stress induced by ROS, (ii) physical disruption of microbial membranes, (iii) genotoxic effects and protein inhibition arising from intracellular interactions, and (iv) inhibition of bacterial adhesion and biofilm formation. While these mechanisms are discussed separately for clarity, they often occur simultaneously and synergistically, contributing to the overall antimicrobial performance of the material.

In the case of nanoparticles, geometry dictates the mode of interaction. While spherical NPs offer high adhesion and surface area, rod‐shaped NPs can exert mechanical pressure, puncturing membranes more effectively due to their elongated structure and high curvature. Higher concentrations generally improve microbial kill rates, although the relationship is not always linear. Excessive concentrations can lead to saturation and aggregation, diminishing efficacy. Regarding charge, cationic (positively charged) NPs are typically more effective than neutral or anionic ones. This is due to the strong electrostatic pull toward the negatively charged bacterial surface, which facilitates easier uptake and more rapid membrane collapse [131, 132].

Another aspect to be taken into consideration is the chemical speciation and ion release kinetics. For instance, when metal or metal oxide (M/MO) nanoparticles are introduced into physiological environments, they undergo complex transformations, including surface oxidation and dissolution. The rate of ion release is governed by environmental factors such as pH, ionic strength, and the presence of organic ligands (e.g., proteins or amino acids), which can form stable complexes with the released ions.

Exogenous ions could compete with essential metals (such as Fe or Mn) for the active sites of enzymes. Once the native metal is displaced by the toxic ion, the enzyme loses its catalytic function, leading to a breakdown in metabolic and signalling pathways [133]. While free metallic ions are often responsible for immediate toxicity, their speciation, whether they remain as free ions or form insoluble precipitates, dictates their long‐term biological impact. For instance, in acidic microenvironments typical of infection sites, the accelerated dissolution of ZnO leads to a localized surge in ion concentration, enhancing the bactericidal effect through a ‘Trojan Horse’ mechanism. Similarly, Cu^2+^ and Cu^+^ ions released from printed copper oxide nanoparticles interact with phosphorus‐ and sulfur‐containing biomolecules (DNA, proteins), causing structural damage and metabolic dysfunction [134, 135]. Zn^2+^ ions from ZnO interact with thiol groups of membrane proteins, causing depolarization and disruption of cellular integrity. Zinc speciation varies considerably as a function of the medium: in acidic environments typical of infection sites, Zn^2+^ release is favored, while at physiological pH, less soluble complexes may form [136, 137]. Ag^+^ ions exert toxicity through interaction with cell membranes, protein denaturation, and interference with ATP synthesis. AgNPs demonstrate particular efficacy against Gram‐negative and Gram‐positive bacteria, fungi, and certain viruses, while maintaining relatively low cytotoxicity toward eukaryotic cells at appropriate therapeutic concentrations [138].

### 5.1 Oxidative Stress and Reactive Oxygen Species Generation

Several antibacterial therapies rely on the production of oxidative stress by ROS. Their bactericidal effect arises from the ability of redox‐active surfaces to generate highly reactive intermediates, such as hydroxyl radicals, superoxide anions, and hydrogen peroxide, which induce oxidative stress and compromise multiple cellular targets simultaneously. Lipid peroxidation, protein oxidation, enzyme inactivation, and nucleic acid damage collectively disrupt essential bacterial functions, ultimately leading to cell death [139, 140].

Many transition metals and their oxides possess potent redox capabilities, enabling them to accept electrons from reactive donor sites. When these materials are incorporated into printed nano‐ and microstructured architectures, redox activity is no longer expressed in homogeneous environments but becomes localized within confined interfacial regions defined by geometry, surface chemistry, and transport limitations. Under these conditions, interactions between metal or metal oxide domains and bacterial cells promote ROS generation with spatially heterogeneous intensity, shaping oxidative stress at the bacteria–material interface rather than in the surrounding bulk medium [140, 141].

For ZnO‐based systems, photocatalysis provides an additional antimicrobial pathway. Under UV irradiation, electron–hole pairs are generated and subsequently react with oxygen and water to produce ROS [137]. The photocatalytic activity of TiO_2_, particularly in its anatase crystalline phase, has been extensively documented for disinfection applications. In printed and microstructured TiO_2_ materials, defect populations and nonstoichiometric surface states introduced during fabrication enable antimicrobial activity under visible light or standard indoor LED illumination, rather than requiring potentially harmful UV exposure, significantly expanding practical applicability [142, 143].

Beyond photocatalytic ROS generation, light can be exploited more broadly within photodynamic therapy (PDT), in which photosensitizer molecules activated by specific wavelengths generate singlet oxygen and other cytotoxic ROS that induce oxidative damage to bacterial membranes, proteins, and nucleic acids. A wide range of photosensitizers have been investigated in antibacterial applications, including porphyrins, phthalocyanines, conjugated polymer nanoparticles, and inorganic hybrid systems combining metal oxide photocatalysts with plasmonic nanostructures. Among the latter, TiO_2_‐based platforms decorated with gold nanorods have demonstrated visible‐light‐driven photodynamic antibacterial activity under blue light irradiation, with quantified oxidative damage to both lipid membranes and bacterial DNA [118]. In parallel, photothermal therapy (PTT) has emerged as a complementary approach, exploiting the capacity of photothermal agents such as plasmonic nanostructures [144], black phosphorus, and carbon‐based materials to convert near‐infrared (NIR) irradiation into localized heat, inducing membrane disruption and protein denaturation at bactericidal temperatures. The combination of PDT and PTT within a single printed platform has shown significant synergistic potential, as the two mechanisms target bacterial cells through distinct and simultaneous pathways, substantially reducing the probability of adaptive resistance emerging compared to single‐mechanism treatments [145, 146]. In 4D‐printed systems, NIR irradiation can additionally serve as an external trigger for shape or permeability changes in the construct, coupling the phototherapeutic response with architectural reconfiguration within a single activation event [36].

### 5.2 Disruption of Microbial Surfaces and Cell Membranes

Physical membrane disruption occurs through the accumulation of metallic nanoparticles on bacterial surfaces. Electrostatic interactions, facilitated by the attraction between positively charged ions and the negatively charged lipopolysaccharides (LPS) in Gram‐negative bacteria or teichoic acids in Gram‐positive species, and mechanical stress alter cell membrane permeability. This interaction often induces lipid peroxidation, where ROS‐mediated attacks on membrane phospholipids compromise structural integrity. Consequently, vital intracellular components such as proteins and ions leak out, leading to a fatal chemical imbalance and cell death [131, 133]. Electron microscopy studies have documented “pitting” effects, pore formation, and structural lesions in membranes of *E. coli* and *S. aureus* exposed to CuO and ZnO doped with Fe or Mn. Interestingly, the thicker peptidoglycan layer of Gram‐positive bacteria can sometimes act as a partial diffusion barrier, delaying nanoparticle penetration, compared to the thinner, LPS‐rich outer membrane of Gram‐negative strains [147, 148]. High‐resolution printing techniques such as aerosol jet printing can create “nano‐spike” or high‐roughness surface morphologies; when bacteria colonize these surfaces, mechanical tension on their membranes leads to physical rupture, a process termed mechanocidal activity [5, 149]. Perturbation of the transmembrane potential compromises vital functions, including nutrient transport, electron transport chain activity, and ATP synthesis. Metal ions can additionally interfere with essential nutrient acquisition systems (including iron and zinc uptake), creating lethal nutrient deficiency states in microorganisms [150].

### 5.3 Genotoxicity and Protein Inhibition

Another bactericidal pathway of metal and metal oxide (M/MO) nanoparticles involves their interference with DNA and cytosolic enzymes across various microorganisms. Even though the proper mechanism is still unclear, metals and their ions are proven to be able to physically interact with DNA in an aspecific way [151], often leading to DNA condensation, which physically obstructs the replication machinery. Furthermore, ROS‐induced base damage causes mutations and strand breaks. These factors collectively block DNA replication and transcription, preventing the cell from synthesizing life‐sustaining proteins.

Additionally, these metals and ions bind directly to enzymes, particularly targeting the thiol groups (−SH) of cysteine residues within active sites. This effectively shuts down metabolic and signalling pathways, altering their tertiary structures and impairing critical processes like digestion and respiration. Furthermore, toxic metal ions can displace essential metal cofactors from metalloenzymes, a process known as “isomorphous replacement,” leading to complete enzymatic dysfunction. Consequently, bacterial cells experience declining metabolic function and eventual cell death. Specific metal ions, including copper(I), zinc(II), manganese(II), and iron(II), have been documented to operate through this antibacterial mechanism [152].

### 5.4 Adhesion and Biofilm Formation Inhibition

Most bacteria rely on the formation of biofilms, composed of microorganisms and polymeric extracellular matrices, that tightly adhere on abiotic and biotic surfaces. This multicellular lifestyle allows for enhanced survival, horizontal gene transfer, and the chronic persistence of infections [153]. Preventing the colonization of surfaces and materials, especially if intended for medical applications, represents a critical strategy to limit the emergence and selection of antibiotic‐resistant strains that may arise under prolonged antibiotic selective pressure. As shown previously, TiO_2_ coating was used to create a surface on which *S. mutans* adhesion was severely reduced by altering surface energy and wettability, making the environment energetically unfavourable for bacterial “docking” [4]. Several M/MO nanoparticles can interfere with Quorum Sensing (QS) pathways, disrupting the chemical signalling for biofilm maturation. They can also penetrate and degrade the EPS matrix by generating localized oxidative stress or acting as “*nanozymes*” that break down structural polysaccharides. This increases the susceptibility of internal persister cells to both metal ions and conventional antibiotics [154].

## 6 Stability, Ageing, and Translational Challenges

The antimicrobial effect of printed materials and microstructured materials results from a dynamic chemical equilibrium that constantly changes during the production process, storage, as well as use. The availability of reactive agents depends on a balance of molecular stability, degradation of materials through chemical reactions, leaching of agents, and interaction of agents with the surrounding matrix, switching from an initial “burst release” for immediate disinfection and a “sustained release” for long‐term efficacy. Moreover, this equilibrium is heavily influenced by environmental factors such as humidity, temperature, and UV exposure, which can trigger oxidative ageing or surface passivation. Consequently, the interaction of agents with the surrounding matrix and the ambient atmosphere must be carefully engineered to prevent premature degradation and preserve their biocidal efficacy [3, 155].

### 6.1 Physicochemical Stress During Fabrication and Use

Right from the beginning of the fabrication process, printed and microstructured antimicrobial materials find themselves subjected to a multitude of physicochemical stresses: thermal, photochemical, oxidative, and mechanic stimuli [156, 157]. The curing process and strong electric fields during electrospun nanofiber synthesis, solvent removal during inkjet printing, and the scale confinement during DPN introduce strongly reactive microenvironmental conditions [158]. These conditions can lead to a subtle change in the chemical state of the embedded antimicrobial molecules, peptides, or metal‐based compounds, thereby causing partial oxidation, photodegradation, or conformational changes [3, 26]. This means that at the time of deployment, the chemical state can be already profoundly different from the pristine state, thereby underscoring the critical need to consider such early‐stage chemical changes associated with material fabrication for the effective development of functional antimicrobial platforms.

### 6.2 Long‐Term Chemical Ageing of Structured Materials

After fabrication, structured materials also undergo continuous changes at the molecular and chemical levels. For example, polymers experience slow relaxation, oxidation, hydrolysis, scission, and the development of reactive radicals, whereas smaller molecules/peptides can diffuse, aggregate, and potentially react with the surrounding material [159]. These processes are generally accelerated for confined and highly porous material structures because of greater interfacial areas and slower diffusion rates, resulting in complexities and nuances for molecular stability, release rates, local concentrations, and the availability of antibacterial agents, even in the absence of obvious macroscopic changes [3, 26, 159]. In terms of molecular processes, these ageing phenomena are generally linked to the progressive development of secondary functional groups, radicals, and changes to molecular mobility and/or crystallinity, all prior to observable changes to the material structure and correlating to direct links between material and function [159]. These processes at the early stages of ageing can, in principle, be detected only by very sensitive analytical methods, which have to focus on the ability to detect specific radical intermediates and changes to functional groups during the early stages of the ageing process for polymers. Methods such as electron spin resonance spectroscopy, as well as fluorescence‐based approaches including probe‐assisted methodologies, have served as early warning systems to indicate ageing well before chain scission reactions, crosslinking, or macroscopic degradation. In general, long‐term ageing of polymeric materials with potential antimicrobial properties could help to compromise this long‐term purpose [159].

More fundamentally, however, long‐term changes to surface chemistry, release rates, or localized concentrations of antimicrobial agents as driven by material ageing could logically contribute to changes that cumulatively favor adapting bacteria that are capable of distinguishing between lethal versus sublethal doses, directly correlating polymer ageing to AMR [160]. At this juncture, it is reasonable to conclude that increased absorption of copollutants and biofilm formation could exert shear localized‐inhibitory effects that cumulatively favor adapting microbes with increased resistance to antibiotics. As such, polymer ageing in either antimicrobial materials or in long‐lived polymers in the natural world presents such an important yet overlooked process that directly relates to how AMR is dispersed.

### 6.3 Chemical Interactions at the Bacteria–Material Interface

At a biointerface, antimicrobial printed and microstructured polymers readily develop a conditioning film consisting of proteins, extracellular polymeric substances, and lipids adsorbed from the surrounding biological medium. This interfacial biochemistry fluctuates in a dynamic environment, influencing the bioavailability, distribution, and ability to react of antimicrobial agents, which is due to noncovalent forces like electrostatic attraction, hydrogen bonding, van der Waals forces, as well as hydrophobic interactions. In varying levels of bacterial envelope components, biofilm age, and environmental conditions, bioavailability might work synergistically to boost antimicrobial agents in binding to a bacterial cellular wall, but in other circumstances, a reduction in effectiveness might be realized due to sequestration, shielding, or competitive binding [137].

Bacterial adhesion to material surfaces is first initiated by long‐range physico‐chemical forces like Lifshitz‐van der Waals and electrostatic interactions, then it is stabilized by short‐range, highly specific molecular interactions. Unlike inert colloidal particles, bacterial cells display a heterogeneous and adaptable outer envelope made of peptidoglycan, teichoic acids, LPS, surface proteins, and polysaccharides whose composition and conformation can change in response to environmental stimuli [161]. These surface macromolecules act as functional ligands capable of mediating both reversible and irreversible attachment to conditioned material surfaces [162]. Material surface properties, including surface charge, polarity, hydrophobicity, roughness, and chemical functionality, critically modulate these interactions, not only by directly influencing bacterial adhesion forces but also by determining the composition, structure, and conformation of the adsorbed biomolecular layer [162, 163]. In this respect, the role of the conditioning film becomes that of a chemical and biological mediator in the interaction between bacteria and the surface material, often dominating the original surface nature of the material. As a result, antimicrobial substances in polymeric matrices are predominantly affected by this interface layer rather than the bacterial cell membrane per se [164]. As biofilm development progresses, the accumulation of extracellular polymeric substances (EPS) profoundly modifies the local chemical microenvironment by increasing matrix density, heterogeneity, and the availability of reactive functional groups. The EPS interface layer then serves as a pool for additional binding sites as well as a diffusion barrier that could inhibit the penetration of antimicrobial material [165, 166]. Besides interfering with the transport of antimicrobials, EPS plays another important role in acting as a protecting barrier that cushions the bacterial cells against environmental stresses such as extreme pH, temperature fluctuations, and high salinity [166]. On the other hand, environmental factors trigger adaptive bacterial surface chemistry remodeling in terms of charge patterns, hydrophobicity, and adhesion‐associated macromolecules, resulting in varied interactions with surface‐bound antimicrobials. In summary, the chemical interaction occurring at the bacteria/material interface is the result of the interaction between the adaptability of the bacteria at the surface, the chemical properties of the material surface, and the composition of the adsorbed biomolecules. Moreover, understanding the mechanisms governing bacteria–material interaction is essential for the rational design of antimicrobial surfaces that prevent adhesion.

### 6.4 Stability of Antimicrobial Function Under Operational Conditions

Environmental and operational factors such as pH, humidity, enzymatic activity, exposure to light, and mechanical stress further refine the chemical microenvironment around antimicrobial constructions. In responsive systems, these factors will purposefully initiate release and changes, whereas in passive constructions, these will contribute to overall chemical ageing. This point draws attention to the idea that the chemical lifetime of these agents does not and cannot necessarily be equivalent to that of the construction concerned, as the integrity of the construction does not necessarily imply continued antimicrobial activity [167, 168]. The aspects related to stability and ageing should be treated as primary design criteria in chemical design instead of being considered only as second‐order parameters. The approach involving molecular confinement, interface stabilization, or the design of microenvironments can be implemented to retain efficacy, avoid unplanned release events, and increase the functional lifespan [168, 169]. The methodology involving the utilization of chemical stability as a parameter in design instead of a weakness enables printed and microstructured materials to be designed for sustaining reliable and strong antimicrobial properties [55, 170, 171].

### 6.5 Scalability, Biosafety, and Regulatory Considerations

Despite the remarkable progress in the design and fabrication of printed antimicrobial platforms, several critical barriers continue to limit their translation from laboratory proof‐of‐concept to clinical and industrial application. A fundamental challenge lies in the trade‐off between printing resolution and scalable manufacturing. High‐resolution techniques such as inkjet printing and DLP achieve feature sizes in the range of 2–80 μm, enabling precise spatial control over antimicrobial agent distribution, but at throughputs that are incompatible with large‐scale production. Extrusion‐based methods offer higher fabrication speeds but at the cost of reduced resolution and greater variability in the spatial distribution of active agents. This inherent trade‐off between precision and throughput remains a major obstacle to the clinically relevant implementation of printed antimicrobial constructs, and its resolution will require the development of printing platforms capable of maintaining chemical fidelity across scales [172].

A second critical gap concerns the in vivo metabolic and degradation kinetics of multicomponent printed platforms. While in vitro release profiles and antimicrobial activity are routinely characterized, the behavior of these materials under physiological conditions remains poorly understood. The degradation of polymer matrices, the speciation and clearance of released metal ions, and the fate of encapsulated peptides in the presence of enzymatic activity, fluctuating pH, and immune responses represent key unknowns that must be addressed before regulatory submission [55]. Sublethal concentrations generated by in vivo degradation kinetics that differ from those measured in vitro represent a particularly underappreciated risk, as discussed in the preceding sections. Finally, the lack of harmonized regulatory frameworks specific to printed antimicrobial materials represents a structural bottleneck. Existing standards provided by ISO and ASTM International predominantly address conventional fabrication methods, leaving multimaterial and stimuli‐responsive printed platforms largely outside the scope of current guidelines [173, 174]. The development of standardized protocols for the characterization of release kinetics, antimicrobial efficacy, and long‐term biosafety under operationally relevant conditions is an essential prerequisite for the clinical translation of this class of materials.

## 7 Summary and Outlook

Additive manufacturing can be a precious tool in the hands of chemists to prepare innovative platforms that exert antimicrobial activity. In this context, printing methods offer a valuable route to the fabrication of functional antimicrobial materials due to their simplicity in fabrication, versatility in design, ability to work with flexible substrates, and precise control over the electrical, optical, and magnetic properties of printed materials. Addressing AMR requires approaches that extend beyond conventional drug delivery to focus on the rational design of chemically engineered interfaces. As highlighted throughout this Review, printing technologies function not merely as fabrication tools but as platforms for chemical design, enabling precise spatial and temporal control over antimicrobial activity. From nanoscale patterning capable of defining highly confined reactive domains to mesoscale and macroscale constructs that regulate molecular transport, release kinetics, and mechanical interactions, structured materials emerge as a unifying framework for antimicrobial design [175, 176]. Starting from DPN's nanoscale precision to droplet‐based microreactors, electrospun fiber mats, and multiscale 3D and 4D printed constructs, structured materials redefine antimicrobial effectiveness as a property emerging from confinement, controlled transport, defect chemistry, and interfacial reactivity. Electrospinning occupies a prominent role within this context, producing nanofibrous scaffolds with high surface area‐to‐volume ratios that facilitate the efficient encapsulation and sustained release of antimicrobial agents, including metal nanoparticles, AMPs, and natural compounds effectively.

Across all scales, antimicrobial performance is shaped by how the microenvironment at the bacteria–material interface is modulated. Nanoscale confinement amplifies local reactivity and defect‐mediated ROS generation. Droplet microreactors and coacervate systems leverage molecular crowding and phase separation to stabilize and enhance labile compounds. Fibrous architectures restrict transport while maximizing surface interaction, and 3D/4D printed structures enable hierarchical, time‐dependent regulation of porosity, release profiles, and chemical activation. In this context, fabrication becomes a means of chemical programming. Equally important is the dynamic nature of antimicrobial function. In printed metal oxides, solid‐state characteristics, such as oxygen vacancy density, mixed oxidation states, and surface hydroxylation, govern ion release and redox behavior. Polymer matrices, though versatile and biocompatible, evolve over time through ageing, radical formation, and structural relaxation, which can alter their antimicrobial properties. The stability of confined agents, the development of conditioning films, and bacterial adaptations at interfaces must all be treated as integral design elements, not secondary considerations.

Despite significant advances, key obstacles remain on the path to clinical and industrial application. Reproducibility in printed chemistries, long‐term stability under real‐world conditions, scalability, and the establishment of standardized testing protocols are still maturing. Material ageing must be recognized as a dynamic factor capable of altering release profiles over time, potentially generating sublethal antimicrobial concentrations that may promote the evolution of bacterial resistance. To this end, the long‐term efficacy and safety of any printed antimicrobial platform cannot be assessed independently of its ageing behavior. Future efforts should integrate advanced in situ and *operando* characterization with predictive materials modelling to build reliable structure–property–function relationships.

As a perspective, the fusion of additive manufacturing, responsive polymers, and microfluidic compartmentalization offers transformative possibilities. Programmable antimicrobial microreactors, infection‐responsive 4D architectures, and integrated theranostic platforms point toward adaptive, self‐regulating systems capable of detecting and responding to microbial threats in real time. Such systems could reduce chemical load, minimize systemic toxicity, and ease the selective pressure associated with continuous antibiotic use. Future advances will hinge on the ability to unify precursor chemistry, defect engineering, polymer network design, and microenvironmental control within cohesive structure–property–function frameworks. Promising directions include the intentional stabilization of metastable phases, the programming of redox cycling pathways, and the integration of stimuli‐responsive transformations with regulated transport. These strategies point toward systems in which the local antimicrobial concentration at the bacterial interface is maintained above the MIC while the administered dose is minimized.

Looking further ahead, the design of antimicrobial platforms is increasingly informed by computational approaches that operate upstream of fabrication, enabling the rational generation of molecular candidates prior to any experimental step. Generative AI has demonstrated remarkable capabilities in the de novo design of AMPs, circumventing the limitations of template‐based sequence modification and enabling systematic exploration of combinatorial sequence spaces. Diffusion‐based generative models have proven particularly powerful, producing large libraries of in silico candidates of which experimentally validated subsets have shown broad‐spectrum antibacterial and antifungal activity, including in vivo efficacy against clinically relevant multidrug‐resistant pathogens [177, 178]. Large language model‐based pipelines have further extended this paradigm to high‐throughput sequence mining and generation, yielding novel AMPs with reduced susceptibility to resistance development [179]. In parallel, machine learning algorithms have demonstrated the ability to predict ink printability, viscosity, and drug dose from compositional and rheological descriptors, thereby reducing the empirical burden of formulation development [180]. Together, these advances position generative AI as an active contributor to the rational chemical design of next‐generation antimicrobial platforms, from molecular sequence generation to ink formulation and fabrication. Ultimately, the rational integration of printing technologies, chemical design, and computational tools offers a concrete and scalable strategy toward increasingly effective antimicrobial materials capable of meeting the clinical challenges posed by the global spread of drug‐resistant pathogens.

## Author Contributions

**Giorgia Puleo:** conceptualization, supervision, writing‐original draft, writing – review & editing. **Silvia Orecchio:** writing – original draft, writing – review & editing. **Claudia Pellerito:** writing – original draft. **Vincenzo Piscopo:** writing – original draft, writing – review & editing. **Dario Savoca:** writing – original draft, writing – review & editing. **Vittorio Ferrara:** writing – original draft, writing – review & editing. **Sebastiano Alberto Fortuna:** writing – original draft. **Alessandra Giardina:** writing – original draft. **Bruno Pignataro:** supervision, writing – review & editing. **Paola Costanzo:** supervision, writing – review & editing. **Floriana Campanile:** supervision, writing – review & editing. **Giuseppe Arrabito:** conceptualization, funding acquisition, supervision, writing‐original draft, writing‐review & editing. All authors have approved the final version of the manuscript.

## Funding

This study was supported by Ministero dell’Università e della Ricerca and Università degli Studi di Palermo.

## Conflicts of Interest

The authors declare no conflicts of interest.
